# 芦可替尼联合低剂量后置环磷酰胺预防恶性血液病单倍体造血干细胞移植急性移植物抗宿主病的临床研究

**DOI:** 10.3760/cma.j.cn121090-20230929-00153

**Published:** 2024-02

**Authors:** 小平 李, 玉 李, 林 刘, 忠涛 袁, 佑铖 王, 彦成 董, 定松 张, 静 冯, 应年 陈, 三斌 王

**Affiliations:** 联勤保障部队第九二〇医院血液科，昆明 650000 Department of Hematology, 920th Hospital of Joint Logistics Support Force, Kunming 650000, China

**Keywords:** 芦可替尼, 移植物抗宿主病, 单倍体造血干细胞移植, 环磷酰胺, Ruxolitinib, Graft versus host disease, Haploidentical hematopoietic stem cell transplantation, Cyclophosphamide

## Abstract

**目的:**

评估芦可替尼联合低剂量后置环磷酰胺为基础的急性移植物抗宿主病（GVHD）预防方案在恶性血液病单倍体造血干细胞移植（haplo-HSCT）中的临床疗效和安全性。

**方法:**

收集2022年1月至12月在联勤保障部队第九二〇医院行haplo-HSCT患者，所有患者在预处理后予以减低剂量后置环磷酰胺、兔抗人胸腺细胞免疫球蛋白、芦可替尼、甲氨蝶呤及环孢素A、霉酚酸酯等预防急性GVHD，分析所有患者移植结局、并发症及生存情况。

**结果:**

共纳入52例haplo-HSCT患者，男29例（55.8％），女23例（44.2％），中位年龄为28（5～59）岁。急性髓系白血病25例，急性淋巴细胞白血病17例、骨髓增生异常综合征6例、慢性髓性白血病2例，骨髓增殖性肿瘤2例。98.1％的患者实现正常植入，其中Ⅱ～Ⅳ、Ⅲ/Ⅳ度急性GVHD发生率分别为19.2％（95％ *CI* 8.2％～30.3％）、7.7％（95％ *CI* 0.2％～15.2％）。未发生严重消化道黏膜炎。EB病毒、巨细胞病毒再激活率分别为40.4％、21.3％。9.6％的患者在随访期间复发，移植后1年总生存率、无进展生存率、非复发死亡率分别为86.5％（95％ *CI* 76.9％～96.1％）、78.8％（95％ *CI* 67.4％～90.3％）、11.5％（95％ *CI* 2.6％～20.5％）。

**结论:**

在恶性血液病患者haplo-HSCT中，芦可替尼联合减低剂量后置环磷酰胺为基础的急性GVHD预防方案可有效控制急性GVHD且安全性良好。

## Abstract (en)

**Objective:**

To investigate and verify a novel acute graft versus host disease（aGVHD） prevention protocol in the context of haploidentical hematopoietic stem cell transplantation （haplo-HSCT）.

**Methods:**

Patients who underwent haplo-HSCT in our center between January 2022 and December 2022 were included. All patients received reduced doses of cyclophosphamide, Rabbit anti-human tymoglobulin, ruxolitinib, methotrexate, cyclosporine, and MMF to prevent aGVHD. The transplantation outcomes, complications, and survival rate of all patients were investigated.

**Results:**

A total of 52 patients with haplo-HSCT were enrolled, 29（55.8%） male and 23（44.2%） female, with a median age of 28（5–59） years. There were 25 cases of acute myeloid leukemia, 17 cases of acute lymphocyte leukemia, 6 cases of myelodysplastic syndrome, 2 cases of chronic myeloid leukemia and 2 cases of myeloproliferative neoplasms. 98.1％ of patients had successful engraftment. The incidence of Ⅱ-Ⅳ aGVHD and Ⅲ-Ⅳ aGVHD was 19.2％ （95％ *CI* 8.2％–30.3％） and 7.7％ （95％ *CI* 0.2％–15.2％）, respectively. No patients experienced severe gastrointestinal mucositis. The Epstein-Barr virus and CMV reactivation rates were 40.4％ and 21.3％, respectively. 9.6％ of patients relapsed during followup, with 1-year overall survival, progression-free survival, and non-relapse mortality rates of 86.5％ （95％*CI* 76.9％–96.1％）, 78.8％ （95％*CI* 67.4％–90.3％） and 11.5％（95％*CI* 2.6％–20.5％）, respectively.

**Conclusion:**

Ruxolitinib combined with a low dose of PTCY is a safe and effective first-line aGVHD prevention strategy.

异基因造血干细胞移植（allo-HSCT）是治愈血液系统恶性肿瘤的最终方法之一，急性移植物抗宿主病（GVHD）是移植早期常见的并发症之一。Ⅲ/Ⅳ度急性GVHD通常伴随一系列不可逆的临床表现，是造成移植后非复发死亡的重要原因[1]。目前，国内主流的急性GVHD预防方案为以“钙调磷酸酶抑制剂+霉酚酸酯/甲氨蝶呤±兔抗人胸腺细胞免疫球蛋白（rATG）”为基础的北京方案[2]，国外应用较为广泛的则为以“后置环磷酰胺（PTCY）”作为骨架的急性GVHD预防方案[3]。芦可替尼是选择性Janus激酶（JAK）1/2小分子抑制剂，可通过抑制JAK/STAT信号通路有效降低其下游炎症信号通路，2014年获批治疗骨髓纤维化[4]。近年来已有研究证明芦可替尼对糖皮质激素耐药的急慢性GVHD疗效极佳[5]–[6]，且不影响移植物抗白血病（GVL）效应。本研究拟结合北京方案及PTCY方案的优势，通过调整药物剂量并加用芦可替尼，以寻求并验证一种新的急性GVHD预防方案。

## 病例与方法

1. 病例：本研究纳入2022年1月至12月在我中心行单倍体造血干细胞移植（haplo-HSCT）的恶性血液病患者（排除因新冠病毒感染导致死亡的患者），对其临床资料进行回顾性分析。所有患者均签署知情同意书。

2. 预处理方案：所有患者均采用以下预处理方案：氟达拉滨30 mg·m^−2^·d^−1^×6 d；白消安130 mg·m^−2^·d^−1^×1～2 d（移植前疾病未达完全缓解的患者连续2 d给药，完全缓解的患者给药1次）；美法仑100 mg/m^2^及托珠单抗8 mg/kg（造血干细胞输注前给药）。

3. 急性GVHD预防方案：①环孢素A，移植后5 d（+5 d）开始，起始剂量为2 mg·kg^−1^·d^−1^，+5 d～ +50 d血药浓度（250±50）mg/L，+51 d～+110 d血药浓度（150±50）mg/L，+111 d～+180 d逐渐减停。②环磷酰胺25 mg·kg^−1^·d^−1^，+3 d、+4 d。③甲氨蝶呤5 mg/m^2^，+3 d。④霉酚酸酯15 mg/kg每日3次，+5 d～+34 d。⑤rATG 2.5 mg/kg，植入当天给药（应满足PLT>10×10^9^/L）。⑥芦可替尼5 mg每日2次，移植前1 d（−1 d）～+21 d；2.5 mg每日2次，+22 d～+28 d；2.5 mg每日1次，+29 d～+35 d。

4. 急性GVHD治疗方案：一线治疗为甲泼尼龙1～2 mg·kg^−1^·d^−1^，如治疗无效，酌情应用依那西普、布地奈德胶囊、芦可替尼（如发生Ⅱ～Ⅳ度急性GVHD，芦可替尼加量至10 mg每日2次）、英夫利昔单抗、维得利珠单抗、rATG、间充质干细胞输注等二线治疗方法。出现植入综合征或围植入综合征且血清IL-6浓度大于正常值上限10倍时额外给予一剂托珠单抗8 mg/kg。急性GVHD及慢性GVHD的诊断及分度分别参照西奈山急性GVHD国际联盟标准及西雅图BMT中心标准。

5. 感染防治：所有患者均住百级层流病房，在移植全程给予三唑类或棘白菌素类药物预防真菌、更昔洛韦/阿昔洛韦交替预防病毒、磺胺甲唑预防卡氏肺孢子虫感染，粒细胞缺乏时经验性给予抗生素预防细菌感染。

6. 随访：采用门诊随访、电话随访、查阅住院病历等方式进行随访，随访截止时间为2023年8月10日。中位随访时间为12.0（1.2～19.3）个月。

7. 统计学处理：采用SPSS 26.0软件进行数据统计、分析及绘图。采用Kaplan-Meier曲线法进行生存分析。

## 结果

1. 病例特征：本研究共纳入52例在我中心行haplo-HSCT的恶性血液病患者，男29例（55.8％），女23例（44.2％），中位年龄为28（5～59）岁。急性髓系白血病（AML）25例，急性淋巴细胞白血病（ALL）17例，骨髓增生异常综合征（MDS）6例，慢性髓性白血病（CML）2例，骨髓增殖性肿瘤（MPN）2例。42例（80.8％）患者在移植前处于完全缓解状态，流式细胞术微小残留病（MRD）阴性占40.4％。患者一般资料及疾病情况见表1。

**表1:** 52例接受芦可替尼联合低剂量后置环磷酰胺为基础急性移植物抗宿主病预防方案haplo-HSCT恶性血液病患者的临床资料

指标	结果
性别［例（%）］	
男	29（55.8）
女	23（44.2）
年龄［例（%）］	
<18岁	15（28.8）
18~<50岁	34（65.4）
≥50岁	3（5.8）
诊断［例（%）］	
AML	25（48.1）
ALL	17（32.7）
MDS	6（11.5）
CML	2（3.8）
MPN	2（3.8）
移植前疾病状态［例（%）］	
CR	42（80.8）
非CR	10（19.2）
单个核细胞输注量［×10^8^/kg，*M*（范围）］	10.9（5.0~26.3）
CD34^+^细胞输注量［×10^6^/kg，*M*（范围）］	6.3（4.2~16.5）
供患者性别组合［例（%）］	
女供男	16（30.8）
男供女	17（32.7）
男供男	15（28.8）
女供女	4（7.7）
供患者血型［例（%）］	
相同	22（42.3）
不相同	30（57.7）
供患者HLA相合程度［例（%）］	
6/12	20（38.5）
7/12	12（23.1）
8/12	7（13.5）
9/12	10（19.2）
10/12	2（3.8）
11/12	1（1.9）

注 haplo-HSCT：单倍体造血干细胞移植；AML：急性髓系白血病；ALL：急性淋巴细胞白血病；MDS：骨髓增生异常综合征；CML：慢性髓性白血病；MPN：骨髓增殖性肿瘤；CR：完全缓解

2. 植入情况：中性粒细胞及血小板中位植入时间分别为12（8～26）d、12（9～15）d。1例（1.9％）患者出现原发性植入失败，于移植后28 d更换供者行二次移植并成功植入。血小板及粒细胞植入率分别为98.1％及98.1％。

3. 急性GVHD发生情况：至随访截止，10例（19.2％）患者发生Ⅱ～Ⅳ度急性GVHD，中位发生时间为移植后29（12 ～72）d。其中累及消化道、肝脏、皮肤急性GVHD分别为5、5、2例（1例为肠道合并肝脏GVHD，1例为皮肤合并肠道GVHD）。4例（40.0％）患者糖皮质激素治疗有效，2例（20.0％）患者在糖皮质激素治疗基础上加用二线治疗药物后获得完全缓解。4例（7.7％）患者发生Ⅲ/Ⅳ度急性GVHD，其中1例患者在+18 d出现肠道Ⅳ度急性GVHD，予以糖皮质激素、芦可替尼、布地奈德胶囊、依那西普、维得利珠单抗治疗并获得有效控制，随后患者出现肺感染，肠道GVHD复发，于+204 d死亡。1例患者在+12 d发生皮肤Ⅳ度急性GVHD，随后于+29 d发生肠道Ⅳ度急性GVHD合并肺真菌感染，糖皮质激素、芦可替尼、巴利昔单抗等治疗效果欠佳，于+57 d死亡。1例患者+17 d出现肠道Ⅳ度急性GVHD，随后迅速出现血便，一线及二线抗急性GVHD治疗均无效，于+36 d死亡。1例患者移植后2个月余MRD转为阳性，停用免疫抑制剂后出现不可控制的肠道急性GVHD合并带状疱疹，于+108 d死亡。

4. 移植相关并发症：10例（19.2％）患者发生出血性膀胱炎，其中2例（3.8％）为重度。予以水化碱化、糖皮质激素、左氧氟沙星处理后所有患者均获得完全缓解。1例患者在移植后出现脑梗死，予以阿司匹林治疗后出现消化道出血，予以止血、抑酸、成分血输注后好转。所有患者均未发生肝小静脉闭塞症（VOD）、血栓性微血管病（TMA）及重度消化道黏膜炎。

5. 感染发生情况：6例（11.5％）患者在移植后100 d内出现真菌感染突破，其中3例发生于持续使用糖皮质激素过程中。EBV、CMV再激活发生率分别为40.4％、23.1％，CMV血症患者未使用莱特莫韦预防感染，未发生CMV病及移植后淋巴增殖性疾病（PTLD）。3例患者出现皮肤疱疹病毒感染（2例发生于移植半年后，经积极抗病毒治疗后均有效控制；1例发生于移植后早期，死于急性GVHD）。

6. 慢性GVHD发生情况：至随访截止，11例（21.2％）患者出现慢性GVHD，轻度8例，中度3例，主要累及皮肤（8例）、口腔（4例）、眼睛（3例）、肝脏（3例）及关节（2例）。轻度慢性GVHD予以芦可替尼单药治疗，中度慢性GVHD予以芦可替尼联合糖皮质激素治疗，均控制稳定，未发生肺慢性GVHD。

7. 复发及生存：至随访截止，5例（9.6％）患者出现疾病复发：1例患者移植后3个月全面复发，予以化疗及供者CAR-T细胞治疗后再次缓解，CAR-T细胞治疗14个月后出现MRD转阳复发，放弃治疗后现仍带病生存。1例CML患者移植后4个月后出现全面复发，予以口服奥雷巴替尼后再次获得持续完全缓解。1例患者移植后4个月余复发，复发后行供者CAR-T细胞治疗，获得持续完全缓解。2例患者移植后早期出现MRD转阳，予以化疗联合供者淋巴细胞输注，其中1例患者出现皮肤GVHD、MRD转阴且持续缓解，另1例患者死于不可控的GVHD。移植后1年总生存（OS）率为86.5％（95％*CI* 76.9％～96.1％）（图1A），无进展生存（PFS）率为78.8％（95％ *CI* 67.4％～90.3％）（图1B），非复发死亡率（NRM）为11.5％（95％*CI* 2.6％～20.5％）（4例死于急性GVHD，1例死于感染，1例死于出血）。

**图1:**
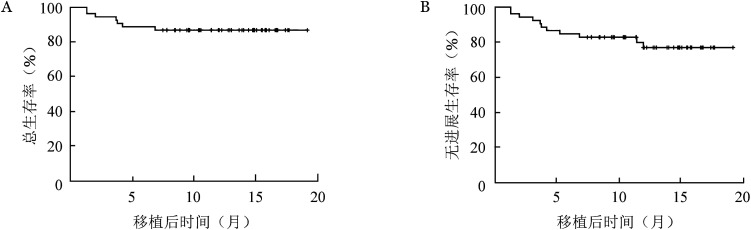
52例恶性血液病患者芦可替尼联合低剂量后置环磷酰胺为基础的急性移植物抗宿主病预防方案单倍体造血干细胞移植后总生存（A）和无进展生存（B）曲线

## 讨论

急性GVHD的预防药物种类繁多，传统药物多以非特异性免疫抑制剂为主。随着新药不断出现，抗体类药物、小分子抑制剂等逐渐应用于临床[7]–[8]。国内外应用较为广泛的急性GVHD预防方案为北京方案及PTCY方案。北京方案在国内开展时间长，应用范围广。在单倍体移植中rATG的用量通常为10 mg/kg，Ⅱ～Ⅳ度急性GVHD及Ⅲ/Ⅳ度急性GVHD发生率分别为43％及14％，慢性GVHD发生率为53％[2]。ATG常见不良反应为高感染风险及造血重建延迟，目前有部分研究对其进行减量。PTCY方案由Johns Hopkins团队首先应用于haplo-HSCT，其主要通过在异体反应性T细胞活化阶段选择性对其进行杀伤，阻断其产生的细胞毒作用、避免受者靶器官损伤，从急性GVHD发病机制的多个阶段阻止GVHD发生[3],[9]。有动物实验发现，在移植后2、3、5 d给予环磷酰胺能成功预防GVHD，但在移植后7 d及以后给药则无效[10]。此外，还有研究表明，PTCY对造血干细胞、静息T细胞及调节性T（Treg）细胞影响小，不影响GVL效应及免疫重建，是较为理想的急性GVHD预防方案[11]，近年来国内不少中心也在尝试应用。移植相关毒性是标准剂量PTCY的主要问题，大剂量环磷酰胺具有心脏毒性，部分老年患者无法耐受。足量的PTCY还可导致免疫恢复阶段发生出血性膀胱炎[12]，使糖皮质激素无法减量。有研究发现，Treg细胞作用的发挥与环磷酰胺剂量相关，过高剂量环磷酰胺可使效应T细胞反弹增强，从而削弱Treg细胞功能，导致更为严重的GVHD发生[13]。

为解决以上不足，国内外多家中心在经典方案基础上探索优化急性GVHD预防体系。北京大学人民医院使用低剂量PTCY（14.5 mg·kg^−1^·d^−1^×2 d）联合rATG（2.5 mg·kg^−1^·d^−1^×4 d）和传统北京方案进行预防单倍体移植急性GVHD的比较研究，发现前者Ⅲ/Ⅳ度急性GVHD发生率较低（5％对18％，*P*＝0.003），Ⅱ～Ⅳ度急性GVHD发生率分别为26％、36％（*P*＝0.14），慢性GVHD发生率分别为30％、44％（*P*＝0.07）；两组患者移植后早期CMV感染发生率分别为74％、30％（*P*<0.001）、EB病毒感染发生率分别为21％、20％（*P*＝0.57）[14]。上海瑞金医院Jiang等[15]使用PTCY及他克莫司联合低剂量rATG（2.5 mg/kg，+15 d及+22 d）预防成人淋系肿瘤患者allo-HSCT后急性GVHD，发现Ⅰ～Ⅳ度、Ⅱ～Ⅳ度急性GVHD发生率分别为（13.0±5.1）％、（9.1±6.1）％，1年累积复发率为（12.8±9.2）％，CMV再激活发生率较高（87.5％），但仅有1例患者在移植后100 d内发生CMV肺炎。本组病例中，我们对传统PTCY方案及北京方案进行整合，同时将PTCY及rATG减量，并在移植前后加用芦可替尼，旨在联合多种有效的急性GVHD预防药物优势，在有效控制急性GVHD的同时降低每种药物的不良反应。本研究结果显示，该方案Ⅱ～Ⅳ度急性GVHD及Ⅲ/Ⅳ度急性GVHD发生率分别为19.2％、7.7％，低于北京方案[2]。1年慢性GVHD发生率为19.2％，且未出现重度慢性GVHD。慢性GVHD发生率较传统方案明显减少，可能与本研究随访时间较短有关。出血性膀胱炎发生率为19.2％，未发生严重口腔黏膜炎、癫痫及心脏不良事件。

芦可替尼是JAK1/2选择性抑制剂，已广泛应用于急性GVHD及慢性GVHD的治疗。其作用机制主要在于抑制JAK/STAT信号通路及炎症因子产生，抑制抗原提呈细胞活化和增殖，还可增加患者体内Treg细胞水平、减少T细胞迁移至GVHD靶器官[16]。一项多中心、开放标签Ⅱ期临床研究显示，对71例糖皮质激素耐药的急性GVHD患者（Ⅲ/Ⅳ度急性GVHD占67.6％）使用至少1个疗程芦可替尼，39例（54.9％）治疗有效，其中19例（26.8％）患者获得完全缓解[17]。皮肤、上消化道、下消化道、肝脏反应率分别为61.1％、45.5％、46.0％、26.7％。此外，多项研究证实芦可替尼对多种血液系统恶性肿瘤及实体瘤有直接抑制作用，有望在治疗GVHD的同时保留GVL效应[18]。Morozova等[19]首先报道在骨髓纤维化患者中使用芦可替尼预防移植急性GVHD，结果显示15 mg/d可减低GVHD发生（Ⅲ/Ⅳ度急性GVHD发生率为15％，慢性GVHD发生率为25％）。亦有小样本研究将芦可替尼用于替代钙调磷酸酶抑制剂作为预防急性GVHD的一线用药并取得良好疗效，但发现血象抑制严重[20]。我们在本组病例预处理期间及移植早期加用短疗程芦可替尼，一方面可有效抑制预处理导致的组织损伤及后续炎症激活，另一方面还能预防急性GVHD，此外亦可发挥抗白血病效应。前期我中心曾尝试移植后持续半年使用芦可替尼预防GVHD[21]，但因持续性造血抑制及潜在感染风险而最终缩短用药时间。本组病例移植后真菌感染突破率为11.5％，CMV及EBV再激活率分别为23.1％、40.4％，且无患者出现CMV病或发生PTLD。

本研究结果显示，在恶性血液病患者haplo-HSCT中，芦可替尼联合减低剂量PTCY为基础的急性GVHD预防方案可有效控制急性GVHD且安全性良好。本研究为单中心单臂临床研究、未设对照组、样本量不够大且随访时间较短，以上结果尚需进一步验证。
